# *In vitro* 3D liver tumor microenvironment models for immune cell therapy optimization

**DOI:** 10.1063/5.0057773

**Published:** 2021-10-04

**Authors:** Maxine Lam, Jose Antonio Reales-Calderon, Jin Rong Ow, Giulia Adriani, Andrea Pavesi

**Affiliations:** 1 Institute of Molecular and Cell Biology (IMCB), Agency for Science, Technology, and Research (A*STAR), Singapore, Singapore; 2 Singapore Immunology Network (SIgN), Agency for Science, Technology and Research (A*STAR), Singapore, Singapore

## Abstract

Despite diagnostic and therapeutic advances, liver cancer kills more than 18 million people every year worldwide, urging new strategies to model the disease and to improve the current therapeutic options. *In vitro* tumor models of human cancer continue to evolve, and they represent an important screening tool. However, there is a tremendous need to improve the physiological relevance and reliability of these *in vitro* models to fulfill today's research requirements for better understanding of cancer progression and treatment options at different stages of the disease. This review describes the hepatocellular carcinoma microenvironmental characteristics and illustrates the current immunotherapy strategy to fight the disease. Moreover, we present a recent collection of 2D and 3D *in vitro* liver cancer models and address the next generation of *in vitro* systems recapitulating the tumor microenvironment complexity in more detail.

## I. INTRODUCTION

Primary liver cancer is the 6th most common cancer and the 4th leading cause of cancer-related mortality globally.^1^ Within liver cancer, hepatocellular carcinoma (HCC) constitutes the majority of cases (75%–85%), followed by intrahepatic cholangiocarcinoma (iCCA, 10%–15%) and other rare subtypes. The main risk factors for HCC are chronic infections, such as hepatitis B virus (HBV) or hepatitis C virus (HCV), heavy alcohol intake, obesity, smoking, and type II diabetes, leading to chronic-inflammatory liver diseases and cirrhosis.^1^

### A. Standard-of-care treatment for liver cancer

The current standard-of-care treatment for early-stage HCC is tumor resection or liver transplantation for patients with poor liver function or tumor recurrence. In the latter case, bridging therapies are often administered. These include selective internal radiotherapy (SIRT), where radioactive material is locally administered intratumorally or transarterial chemoembolisation (TACE), where chemotherapeutic drugs are administered with embolic agents within tumor blood vessels to locally increase drug concentration.^2^ TACE is also frequently provided as a treatment for intermediate-stage HCC with considerable success.^3^

Unfortunately, most patients with HCC are diagnosed at the advanced stage, where a systemic treatment is offered instead (Table I). Due to its complex etiology and high intra- and inter-tumoral mutational heterogeneity, conventional therapeutic agents, such as doxorubicin or platinum derivatives, have had minimal effect on improving advanced HCC outcomes. The first systemic treatment to show improvement in patient outcome was Sorafenib, a tyrosine kinase inhibitor that targets multiple receptor tyrosine kinases, and is now the first-line therapeutic option.^4^ Lenvatinib, another receptor tyrosine kinase inhibitor, was shown to be comparable to Sorafenib and is now included as a first-line therapeutic option.^5^ For patients who do not respond to or develop resistance against Sorafenib, the multi-kinase inhibitors, namely Regorafenib^6^ and Cabozantinib,^7^ as well as monoclonal antibodies, such as the anti-VEGFR2 antibody Ramucirumab,^8^ are approved as second-line therapeutic options.

**TABLE I.** Liver cancer systemic therapies.

	Name	Target	Phase	References
Drugs				
	Sorafenib	Multiple receptor tyrosine kinases	Licensed as 1st line	4
	Lenvatinib	Tyrosine kinase inhibitor	Licensed as 1st line	5
	Regorafenib	Multi-kinase inhibitors	Licensed as 2nd line	6
	Cabozantinib	Multi-kinase inhibitors	Licensed as 2nd line	7
Targeted antibodies				
	Ramucirumab	VEGFR2	III	8
Immunomodulators				
	Nivolumab (Opdivo®)	PD-1/PD-L1 pathway	Licensed as 2nd line	9
	Pembrolizumab (Keytruda®)	PD-1/PD-L1 pathway	Licensed as 2nd line	10
Adoptive cell therapy				
	CAR-T cells	GPC3	I	11
	CAR-T cells	CD133	II	12 and 13
	CAR-T cells	MUC1	I/II	NCT02587689
	TCR-T cells	EpCAM	II	NCT02729493
	CAR-T cells	CD147	Pre-clinical	14
	TCR-T cells	AFP	I/II	NCT03971747
				NCT03998033
	TCR-T cells	NKG2D	Pre-clinical	15
	TCR-T cells	HBV transcripts	Pre-clinical	16

### B. Immunotherapy for liver cancer

Immunotherapy has proven to be highly successful in treating certain refractory cancers and provides an opportunity to target liver tumors that have traditionally had limited therapy options. Clinical trials on immune checkpoint inhibitors (ICIs), such as anti-PD-1 antibodies Nivolumab^9^ and Pembrolizumab,^10^ have shown efficacy and led to the approval of these drugs as second-line therapies for advanced HCC. ICIs work by re-invigorating the patient's anti-tumor immune response, and its success is, therefore, dependent on the tumor immune environment. However, the use of ICIs may not be beneficial for patients whose immune cells have been compromised due to disease or prolonged radio- and chemotherapy.^17^

### C. Adoptive cell therapy development for liver cancer

Another type of immunotherapy, adoptive cell therapy (ACT), seeks to overcome this obstacle by introducing anti-tumor immune cells into the patient rather than relying on the patient's endogenous immune cells. ACT involves the isolation of immune cells (T cells or NK cells) from patients, followed by *in vitro* expansion and genetic modification to include a T cell receptor (TCR) for tumor specificity, and may also include additional alterations to improve immune cell proliferation and persistence, as done for chimeric antigen receptors (CARs). A critical difference between CAR and TCR is that the CAR can only target cell surface antigens, while the TCR can also target intracellular antigens as long as they can be displayed by the major histocompatibility complexes (MHCs).

For immune cells to target tumors with minimal off-target effects, specific antigens need to be identified. The most studied for HCCs is glypican-3 (GPC3), a heparan sulfate proteoglycan over-expressed in HCCs that can be used as a serum biomarker for diagnosis.^18,19^ CAR-T cells against GPC3 can effectively suppress the growth of cell line-based orthotopic xenografts^20^ and subcutaneous patient-derived xenografts.^21^ Further modifications to anti-GPC3 CAR-T cells, such as the removal of PD-1,^22^ the capability to secrete soluble PD-1,^23^ IL-12,^24^ or co-express IL-15 and IL-21,^25^ have improved their killing of HCC cells *in vitro* and *in vivo*. The results of two completed phase I trials were published recently,^11^ indicating some preliminary efficacy of the treatment and the development of cytokine release syndrome in most patients. However, with multiple phase I/II trials still ongoing, the full potential of anti-GPC3 CAR-T cells remains to be evaluated.

As with GPC3, alpha-fetoprotein (AFP) is a strong serum biomarker candidate for HCC^26^ and increases diagnostic sensitivity when used together with GPC3.^19^ Anti-AFP CAR-T cells have displayed anti-tumor activity *in vivo*^24^ and with the discovery of AFP epitopes that MHC can display, attempts at anti-AFP TCR-T cells have shown impressive regression in tumor xenograft models.^27^ Phase I/II clinical trials on anti-AFP TCR-T cells are actively recruiting patients.

In addition to GPC3 and AFP, other notable candidates for CAR-T and/or TCR-T cell therapy are being validated. CAR-T cells against CD133, a surface glycoprotein commonly over-expressed in HCC with prognostic value,^28^ have shown to stabilize HCC in most patients in two phase I/II clinical trials.^12,13^ New York esophageal squamous cell carcinoma 1 (NY-ESO-1) belongs to a group of cancer-testis antigens that are typically and exclusively expressed in the gonads but are upregulated in cancer cells. TCR-T cells against NY-ESO-1 have been tested with success in clinical trials on a number of cancers,^29^ suggesting it can potentially be used for HCC as well. Additionally, preclinical studies have suggested that CD147^14^ and NKG2D^15^ are viable targets for ACT.

In HBV- or HCV-related HCC, viral oncogenesis occurs via insertion mutagenesis and the expression of viral proteins that perturb signaling pathways.^30^ Studies utilizing CAR-T and TCR-T cells targeting viral epitopes have reproducibly reduced tumor growth in xenograft models of HBV- and HCV-related HCC.^31–33^ Following tumor regression in one of two patients infused with the TCR-T cells selected against epitopes produced from short HBV transcripts,^16^ a phase I clinical trial has been initiated.

## II. THE LIVER TUMOR MICROENVIRONMENT (TME)

**FIG. 1.**
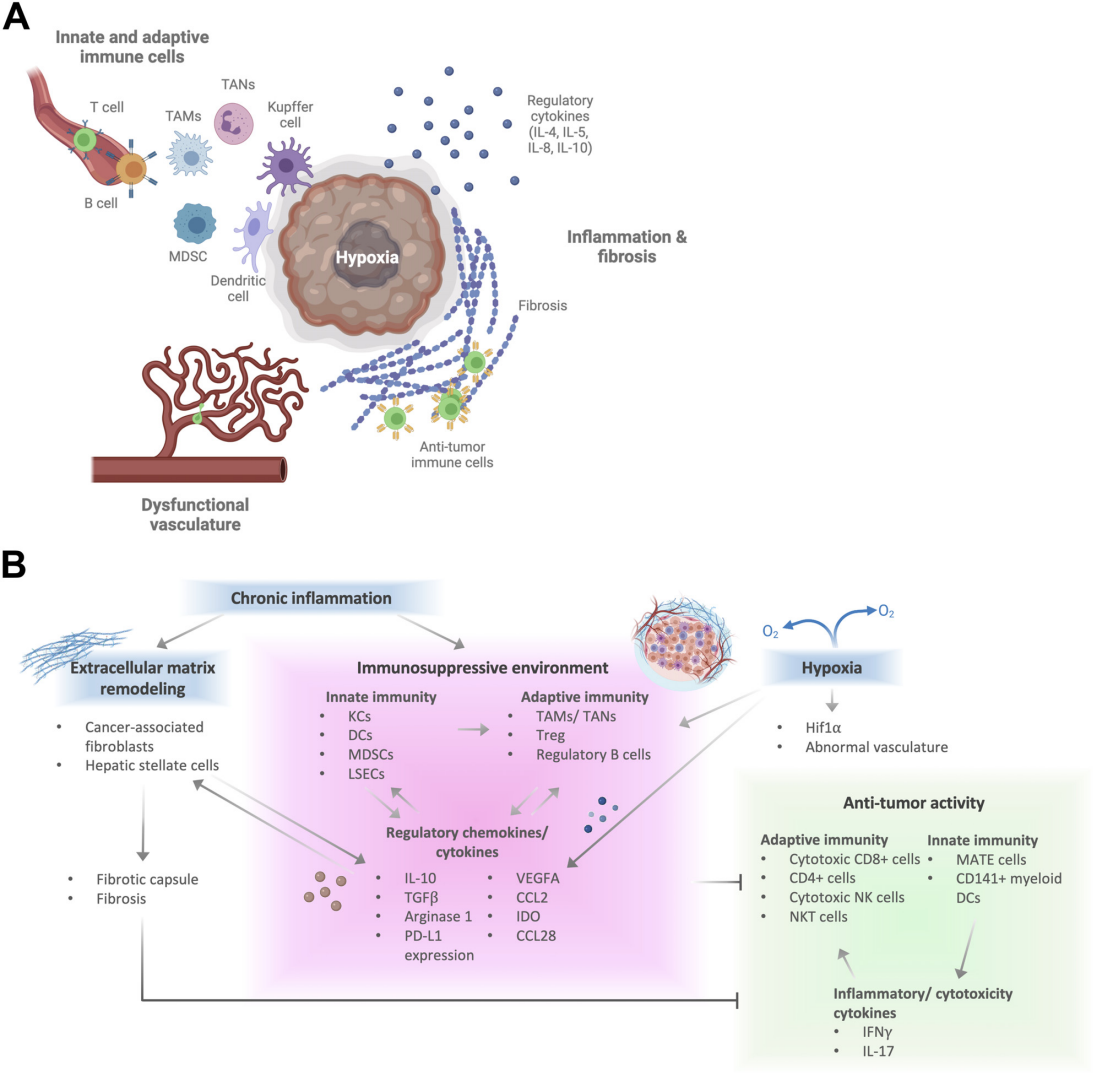
HCC tumor microenvironment components impacting cell therapy. (a) Graphical summary of features of the HCC tumor microenvironment (TME) that influence anti-tumor immunity. The main aspects of the TME that affect cell therapies are inflammation and associated fibrosis, hypoxia and dysfunctional vasculature, and liver innate and adaptive immunity. (b) Details and interactions between aspects of the TME that promote or suppress anti-tumor immunity.

As research into immunotherapy against HCC, such as immune checkpoint inhibitors (ICIs) and ACT, intensifies, it has become increasingly evident that the TME plays an important role in influencing the success of these therapies.^34^ The liver TME consists of endogenous liver cell types, including hepatocytes, stellate cells, and sinusoidal cells, immune cell compartment, the extracellular matrix, and the cytokine/chemokine milieu. The chronic conditions associated with the HCC result in changes to the liver microenvironment that precede and accompany HCC progression. The role of these components in tumor progression has been reviewed extensively in recent years.^35–40^ Critically for immunotherapy, the multiple immunosuppression mechanisms in the tolerogenic liver become dysregulated, leading to the accumulation of immunosuppressive cell populations, defective antigen presentation, and activation of numerous inhibitory receptor-ligand pathways.^41^ Here, we summarize the elements in the tumor microenvironment that can affect the efficacy of ACT against liver tumors (Fig. 1) and briefly discuss ACT strategies for HCC patients.

### A. Inflammation and fibrosis

Despite the difference in risk factors, a common observation across HCC is the presence of chronic cell toxicity, cell death, and inflammation as the liver attempts to resolve these injuries. Additionally, oncogenes activated in HCC can also activate numerous pro-inflammatory pathways such as NF-κ B, IL-6, and TGF β.^42^ Chronic and non-resolving inflammation is, thus, a major feature of HCC and results in the imbalance of pro- and anti-inflammatory cytokines and cell types in the presence of inflammatory mediators, abnormal angiogenesis, and tissue remodeling. Chronic inflammation promotes an immunosuppressive environment in HCC via multiple mechanisms such as the over-representation of immunosuppressive cytokines (IL-4, IL-5, IL-8, and IL-10), promoting M2 macrophage differentiation, the upregulation of inhibitory and downregulation of co-stimulatory signaling by antigen-presenting cells and T cells, and the recruitment of immunosuppressive cell types such as Tregs and MDSCs^42,43^ [Fig. 1(b)].

Chronic inflammation also leads to fibrosis, as inflammatory cytokines activate hepatic stellate cells and Kupffer cells, leading to their production of extracellular matrix (ECM) proteins and matrix metalloproteinases that reorganize the ECM.^43^ The HCC stroma is also enriched for cancer-associated fibroblasts (CAFs), a heterogeneous mix of cells that produce ECM proteins, including collagen.^37^ Excessive fibrosis in and around the tumor also acts as a physical barrier to lymphocyte migration^44,45^ [Figs. 1(a)–1(b)]. Consistent with this, CTNNB1 (gene encoding β-catenin) mutations, which are among the most frequent somatic mutations in HCC, are frequently associated with the formation of a fibrotic capsule around the tumor^46^ and characterized by low lymphocyte infiltration.^46,47^ Additionally, scirrhous HCC, with marked intratumoral fibrosis, has low lymphocyte infiltration. Targeting the ECM component in the TME has been shown to improve the access and efficacy of anti-tumor immune cells,^48–51^ highlighting the role of the ECM in the immune environment.

Additionally, chronic inflammation in HCC via activated NF-κ B signaling leads to leukocyte accumulation and functional immune microarchitecture known as ectopic lymphoid-like structures (ELSs).^52^ ELSs contain a mixture of pro- and anti-inflammatory immune cells and can be pro- or anti-tumorigenic depending on their location within the tumor.^53^ Targeting immunosuppressive ELSs has been shown to improve ICI therapy in an animal model,^54^ illustrating the importance of modulating the immune environment for improving immunotherapy.

### B. Hypoxia and dysfunctional vasculature

The combination of a dense and large tumor with significant fibrosis around it leads to the formation of a nutrient-sparse and hypoxic tumor core.^55^ Hypoxia triggers a cascade of related signaling pathways predominantly acting through hypoxia-inducible factors (HIFs), and HIFs and hypoxia-related gene expression are correlated with poor prognosis in HCC.^56–58^ Hypoxia strongly affects the innate and adaptive immune environment in HCC, acting on Tregs, infiltrating cytotoxic T cells, TAMs, MDSCs, neutrophils, and cancer cells, leading to an overall immunosuppressive environment (previously reviewed^59,60^) Hypoxia also triggers the production of angiogenic factors (such as VEGFs, FGFs, PDGFs, and angiopoietins), leading to tumor neo-angiogenesis and the disruption of normal hepatic vasculature [Fig. 1(b)]. In fact, currently approved first and second-line systemic treatments for HCC target angiogenic pathways^61^ (Table I). Tumor vasculature can inhibit the adaptive immune response to tumors because tumor vasculature can trap immune cells due to their irregular morphology and inconsistent permeability [Fig. 1(a)]. Additionally, tumor-associated endothelial cells can downregulate proteins that promote immune cell extravasation and upregulate proteins that promote the selective apoptosis of effector T cells and proteins that promote the accumulation of immunosuppressive cell types such as Tregs.^62^

### C. Innate and adaptive immune cells

The hepatic macrophages, Kupffer cells (KCs), constitute ∼90% of all tissue macrophages in the body and are key to pathogen capture and immune cell recruitment. KCs are involved in antigen-specific tolerance by producing IL-10, which expands Treg cell populations.^63^ Treg cells and other immunosuppressive cells such as regulatory B cells also increase the expression of IL-10 and further create an immunosuppressive environment.^64^ Dendritic cells in the liver also contribute to the immunosuppressive environment of HCC by recruiting Tregs via the secretion of CCL22^65^ or by activating regulatory B cells that then produce IL-10.^66^ However, in HCC with CTNNB1 gene mutations, the recruitment of pro-inflammatory dendritic cells and, therefore, T cells to the tumor is abrogated by suppressing the production of chemokines CCL4 or CCL5 by tumor cells.^67,68^ Accordingly, immune checkpoint inhibitors were less effective in patients with activated alteration of WNT/β-catenin signaling pathway.^69^

The pro-inflammatory and hypoxic environment found in HCC polarizes macrophages toward a pro-tumor phenotype.^70,71^ These tumor-associated macrophages (TAMs) then produce TNFα, IL-10, and various chemokines, such as CCL17, CCL18, and CCL22, which attract Treg cells.^72,73^ Tumor-associated neutrophils (TANs) have also been found to recruit macrophages and Treg cells.^74^ Myeloid-derived suppressor cells (MDSCs) undergo population expansion during chronic infection with HBV^75^ and are potent producers of IL-10, TGF-β, and arginase that promote Treg cell accumulation and suppress T cell activation.^76^ MDSCs also express the co-inhibitory receptor programmed death-ligand 1 (PD-L1) and can inhibit TCR-mediated T cell activation and proliferation. Accordingly, MDSCs inhibited the cytotoxicity of anti-CEA CAR-T cells^77^ and exogenous cytokine-induced killer (CIK) cells^78^ in mouse models.

### D. Obstacles in adaptive immunity against liver cancer

Adaptive anti-tumor immunity in liver cancer is possible as evidenced by the observation that the co-infiltration of T and B cells into the tumor correlates with a better prognosis.^39^ This is true even for high-grade HCCs such as poorly differentiated HCC that are generally associated with a worse outcome.^79^ Once within the TME, effector lymphocytes secrete a range of factors such as IFN‐γ, CXCL9, CXCL10, and CXCL13, which further recruit B cells.^64,79–81^

As previously mentioned, ACT is a form of immunotherapy that is an active area of research that appears to be having some initial pre-clinical and clinical success. However, HCC remains a complex malignancy that presents unique challenges to ACT. HCC with lymphocyte infiltration constitutes only a minority (10%–25%) of cases.^39^ Tumors, where lymphocyte infiltration is present, can be further sub-grouped into tumors with active immune cells and exhausted immune cells whose behavior appears to be regulated by TGF-β.^82^ Furthermore, chronically inflamed livers due to HCC risk factors, such as viral hepatitis or nonalcoholic fatty liver disease, upregulate inhibitory receptors and cytokines that lead to T cell exhaustion.^41^ Exhaustion in immune cells refers to a hyporesponsive state whereby cells express a higher amount of inhibitory receptors and have a reduced ability in cytokine production and cytotoxicity.^83^ However, this state of exhaustion can be reversed in some cases by interfering with signaling pathways or immune checkpoints.^84^

Since the efficacy of ACT is dependent on their ability to migrate to the tumor and induce cytotoxicity, lymphocyte infiltration and immunosuppressive environments are important factors to consider when choosing the appropriate therapy for patients. In recognition of this, strategies for stratifying patients based on their tumor immune signature have been suggested.^41^ Therefore, ACT may be most effective in tumors where anti-tumor immunity is limited due to immune cell exhaustion or impaired recruitment. For example, engineered cells whose stimulatory signals for activation and proliferation are intrinsic, such as CAR-T or CAR-NK cells, may be particularly effective as they are not dependent on signals from the extrinsic environment which might be immunosuppressive. Alternatively, ACT can be combined with anti-angiogenic drugs such as Sorafenib or checkpoint inhibitors that can reduce the immunosuppressive environment of liver tumors.^85,86^ Strategies for developing ACT, particularly CAR-T therapies, for solid tumors and how to overcome the various barriers to immune cell targeting as well as cytotoxicity, have been extensively reviewed elsewhere.^87^

## III. *IN VITRO* LIVER TUMOR MODELS

The heterogeneity of liver cancer, not just in terms of the tumor-associated antigens, but also in terms of the risk factors and the resulting type of tumor and related microenvironment, highlights the need for patient stratification and therapy personalization to achieve an effective treatment. Likewise, the designing of preclinical models for early-stage ACT trials must consider the complex cell–cell interactions and cytokine/chemokine interactions unique to the subset of liver tumors to adequately recapitulate their specific tumor microenvironment.

**FIG. 2.**
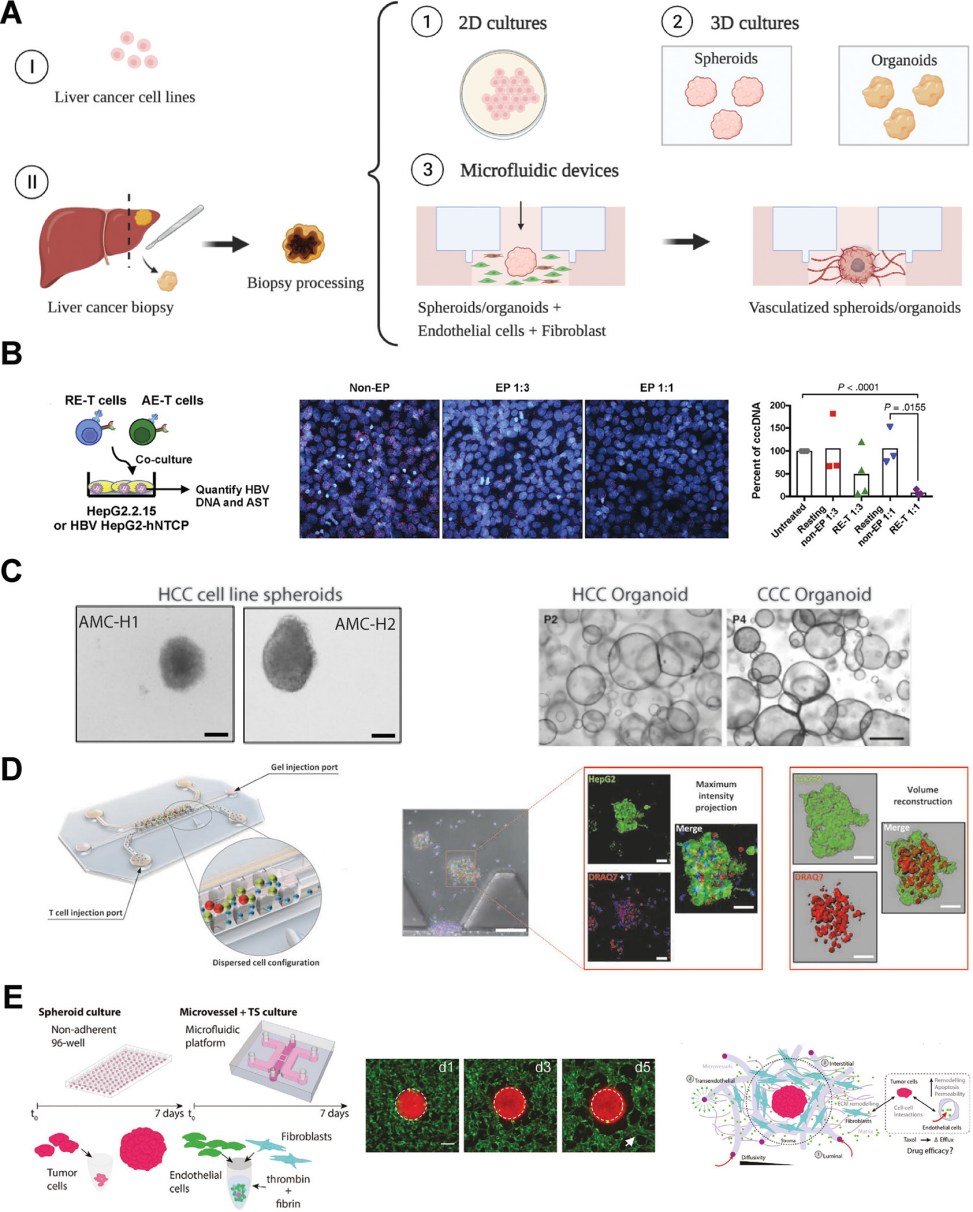
(a) Schematic overview of *in vitro* assays available to study liver cancer tumor immunotherapies. (b) 2D *in vitro* assay developed to study the cytotoxic effect of engineered T cell against HepG2. (c) Examples of hepatocellular carcinoma spheroids and organoids. (d) 3D rendering of a microfluidic device used to study *in vitro* liver cancer immunotherapy (left) and TCR-engineered T cells lyse hepatocellular carcinoma aggregates embedded in the collagen gel of the microdevice (right). (e) 3D vascularized tumor model for cancer-specific characterization and drug dissemination (left), epi-fluorescent images showing the vasculature formation on the tumor within the microfluidic devices (middle), and a schematic summary of diffusive drug transport through the vasculature and TME. Reproduced with permission from (b) Koh *et al.*, Gastroenterology **155**(1),180–193. Copyright 2018 Elsevier.^89^ (c, right) Reproduced with permission from Song *et al.*, J. Exp. Clin. Cancer Res. **37**(1), 109 (2018). Copyright 2018 Author(s), licensed under a Creative Commons Attribution (CC BY) license.^90^ (c, left) Reproduced with permission from Nuciforo *et al.*, Cell Rep. **24**(5), 1363–1376 (2018). Copyright 2018 Author(s), licensed under a Creative Commons Attribution (CC BY) license.^91^ (d) Reproduced with permission from Pavesi *et al.*, JCI Insight **2**(12), e89762 (2017). Copyright 2017 Author(s), licensed under a Creative Commons Attribution (CC BY) license.^92^ (e) Reproduced with permission from Haase *et al.*, Adv. Funct. Mater. **30**(48), 2002444 (2020). Copyright 2020 John Wiley and Sons.^93^

*In vitro* tumor models in cancer research are important screening tools due to their reproducibility and relatively low-cost. There is a tremendous need to improve the available reliable *in vitro* models with appropriate physiological relevance to better understand cancer progression and treatment options at different stages of the disease. The development of more complex *in vitro* systems, with a transition from 2D to 3D models, together with the implementation of biomaterials and microfluidics technologies, has enabled more complex studies that concurrently incorporate several cell types and recapitulate critical spatiotemporal dynamic aspects of the tumor microenvironment.^88^ This section of the review summarizes a collection of liver cancer *in vitro* models, from the simple to the more complex, and their impact on cell therapy studies [Fig. 2(a)].

### A. 2D *in vitro* liver cancer models

Most *in vitro* cancer research studies have been conducted in 2D, using well-characterized cell lines in cell culture dishes. The use of cell lines has several advantages, such as low-cost and population homogeneity, which makes them suitable for high-throughput screenings with highly reproducible and consistent results. HepG2 is the most commonly used liver cell line for *in vitro* studies and provides a model for liver cancer not infected with hepatitis virus.^94^ Huh7 is the second most used liver cancer cell line, and it is recommended to study liver cancer related to hepatitis C infection.^95^ Other cell lines used in liver cancer research are SNU449, Hep3B, HepaRG, BEL-7402, SKHep1, and SMMC-7721, among others.

2D models have been useful for quick assaying of tumor cell death, by simply adding potential chemotherapeutics or cell-based therapies to HCC cells cultured in standard plasticware.^96^ 2D systems are also useful as a starting point for studying molecular interactions between cell types involved in liver cancer that occur via direct cell–cell interaction or by secreted soluble factors. For example, direct co-culture of patient-derived HCC cells with autologous peripheral blood mononuclear cells (PBMCs) revealed that HCC cells, but not normal liver cells, triggered MHC II expression in both HCC cells and CD8^+^ T cells isolated from PBMCs.^97^ More complex interactions with more cell types can also be explored as well. Using this approach, it has been reported that B7-H1 (PD-L1) expression on cultured macrophages^98,99^ or Kupffer cells^100^ with CD8^+^ T cells inhibited T cell proliferation, cytokine production,^100^ and subsequent killing of HCC cells.^98,99^ These findings match the observation that PD-L1^+^ cells are found in histology samples of HCC and correlate with a poorer prognosis, suggesting a potential mechanism for the inhibition of anti-tumor immunity.

Paracrine signaling can also be studied in 2D systems, where cells are separated during culture, such as with the use of a Transwell^®^ insert, but soluble factors can be exchanged in the media. In this way, Hoescht *et al.* found that HCC patient-derived MDSC inhibited autologous NK cell cytotoxicity and cytokine secretion when co-cultured together, but not when they were cultured in separate wells.^101^ Using blocking antibodies, they identified that the cell–cell interaction via NKp30 was required for MDSC-mediated inhibition of NK cell cytotoxicity.^101^ In contrast, Wan *et al.* found through co-culture using Transwells^®^ that TAMs secrete IL-6 to promote HCC stem cell growth.^102^ The use of conditioned media for cells in culture can also shed light on paracrine signaling, and this approach has been used to identify pro-tumorigenic factors secreted by myofibroblasts^103^ and activated hepatic stellate cells.^104^

However, other features of HCC, such as dysfunctional vasculature and fibrosis are impossible to study in 2D. Additionally, cell phenotype can significantly change from 2D to 3D due to changes in cell shape polarity, interactions with ECM proteins, and distribution of biochemical signals and nutrients,^105,106^ resulting in changes in tumor–immune cell interactions (reviewed in Ref. 107).

In studies that evaluate the anti-tumor efficacy of cytotoxic cells toward liver cancer cells, 2D assays have shown different results compared to *in vitro* 3D assays^89,92^ in part because the interaction of T cells and tumor cells in 2D is mediated by gravity rather than longer-range cytokine interactions [Fig. 2(b)], increasing the likelihood of cell-mediated tumor killing. Whereas in the body, T cells must migrate to their target and encounter multiples obstacles and cell types that interfere with their cytotoxicity. Illustrating this point, Lee *et al.* found that engineered T cell cytotoxicity toward HepG2 cells was not impaired by the presence of monocytes in 2D, despite evidence of reduction in the T cell effector function due to the monocytes. However, when the same experiment was carried out in 3D, T cell cytotoxicity was impaired.^108^

### B. 3D *in vitro* liver cancer models

Culturing cells in 3D can replicate some of the cell biology, dynamics of cell–cell interactions and physical obstacles during cancer therapy, and are better at predicting cell behavior and response to therapy *in vivo.*^89,92,109^ Embedding of HCC cells dispersed in ECM such as collagen or Matrigel allows for the studying of single cancer cell proliferation and invasion in 3D and the effects of factors such as ECM stiffness,^110^ nutrient gradients, and other cell types^108,111^ on these read-outs.

However, a key feature of tumors *in vivo* is their significant mass, which leads to the formation of a necrotic and hypoxic core and different zones of cell proliferation and invasion. To address this, liver cancer cell lines can be cultured as aggregates (also termed spheroids because of their morphology) under certain culture conditions, such as in matrix-free suspension or matrix-supported culture.^112^ This 3D organization of cells allows for detailed studies into the cell–cell and cell–ECM interactions, reminiscent of the *in vivo* tumor architecture^113^ as well as oxygen, nutrients, cytokine/chemokine and metabolic gradients.^109,114^ For example, the core of tumor spheroids has more quiescent, hypoxic, and necrotic cells due to the lack of oxygen and nutrients from the medium, while the outer layers have more proliferating cells.^115^

The interactions between different cell populations in the liver tumor can also be investigated by the co-culturing of multiple cell types during spheroid formation^90^ [Fig. 2(c)]. By forming spheroids from HCC cells and stellate cells (LX2), Khawar *et al.* found that liver stellate cells promoted drug resistance to Sorafenib and drove ECM-based migration.^116^ Meanwhile, the addition of endothelial cells during the formation of Huh7 spheroids promotes anti-cancer drug resistance (doxorubicin and sorafenib), mimicking the drug resistance observed in solid tumors.^117^ Such co-culture spheroid models have also been used to study specific cancer development stages, from angiogenesis to migration and invasion, genotoxic potential of compounds, and potential targets for new drugs and cell therapies.^90,118–120^

Because of their ease of formation and scalability, automated high-throughput screenings are also possible with 3D spheroids, speeding up the discovery of new drugs or cell therapies for liver cancer. Liao *et al.* developed a technique to investigate drug sensitivity in cell line and patient-derived HCC spheroids in agarose in a 96 well plate format.^121^ It is worth noting that this study, as well as others, have found that cells in 2D assays exhibit vastly different sensitivities to drugs and cell therapies compared to 3D assays.^117,121^

Liver organoids are 3D physiological *in vitro* structures that can be derived from patient biopsies or from pluripotent stem cells and recapitulate morphological and functional features of *in vivo* tissues, preserve inter-individual features, and maintain the genetic heterogeneity and drug sensitivity of the original tissue.^91^ Patient-derived organoids are obtained by dissociating tumor tissue into single cells, which then self-assemble, forming “mini-tumors” that recapitulate tissue architecture and heterogeneity of the original tumor.^122^ Patient-derived organoids contain multiple cell types that better reflect the biochemical and genomic heterogeneity of the original tumor compared to cell line-derived spheroids and are useful for the identification of personalized therapy regimens.^90,121^ Although organoids derived from cancer tissues have been widely used, there remains a lack of studies with liver cancer-derived organoids as a model.^91,123^ Of the few studies, patient-derived liver cancer organoids have been used to study chemoresistance and drug sensitivity as well as the relationship between gene mutations and drug sensitivity.^91^ Using HCC organoids, Nuciforo *et al.* found that sorafenib reduced HCC organoid growth in a dose-dependent manner, however they could not compare the results with the clinical response as those patients from the organoids were generated and were not treated with sorafenib.^91^ These studies highlight the use of patient-derived organoids as a useful drug discovery tool that incorporates inter- and intra-tumor heterogeneity, which might better reflect the variability in patient response to various therapies and serve as tools for the identification of genes and proteins linked to responders vs non-responders.

While the use of cancer spheroids and organoids for drug screening, genomics investigation, and drug interaction studies are gaining traction, few studies use these models to investigate cell therapies. Busse *et al.* used a 3D *in vitro* model to study the recognition of tumor-associated antigens (TAA) in tumor spheroids by T cells and showed that T cells did not target colon, pancreas, or breast cancer spheroids due to the downregulation in the HLA expression in 3D,^124^ highlighting the importance of incorporating 3D cell organization in early pre-clinical cell therapy studies. Additionally, given the significance of fibrosis in HCC and the link to therapy resistance and immune cell infiltration, *in vitro* studies able to recreate the dense ECM surrounding tumors for the study of drug and cell therapy development will be particularly useful.

Even though 3D spheroid models are more similar to the *in vivo* tumor compared to cells in 2D culture, they often lack an immune and stromal component that limits studies to test interactions between immune or stromal cells and tumor cells. Even with patient-derived samples, which may contain such immune and stromal components, one major challenge is the optimization of tissue, and hence, TME, maintenance after surgical resection. Current methods of biopsy extraction, preservation and patient-derived tissue or cell line maintenance are varied and often result in the loss of significant TME populations.^125^ Efforts to culture patient biopsies containing tumor cells and the cells in the TME are very promising^125–129^ and will likely pave the way for highly physiologically relevant patient-derived complex 3D models.

Patient-derived xenografts (PDXs), where patient-derived tumor cells are cultured within an explant in nude mice, are useful models that partially address these issues, as they preserve more cell types found in the original tumor, and as a likely result retain major histological and genomic features.^130^ Accordingly, there have been increasing efforts to use PDXs in pre-clinical drug testing and biomarker discovery, as they appear to closely resemble clinical patient disease and therapy response.^131^ However, even in PDX models, there is loss of the immune and vascular component of the TME.^132^ Additionally, PDXs are complicated, very expensive and time consuming due to their long engraftment period, often with a low engraftment rates,^133^ and are therefore not scalable.

## IV. NEXT GENERATION *IN VITRO* MODELS

Microfluidics technology improved the ability of *in vitro* models to mimic the physiological conditions of the tumor by enabling the observation of dynamic cellular interactions in a 3D multicellular culture under fluidic gradients. The wide variety of available microfluidic devices and their ability to be easily modified to test various conditions at scale, make these devices an effective solution to develop complex 3D *in vitro* models for cancer research.^134^ The versatility in the design of the microfluidic devices with different channels and compartments enables precise control of the spatiotemporal distribution of different cell types and of physical and chemical gradients.

Our group has previously developed a 3D multicellular microfluidic assay to analyze the targeting and function of (HBV)-specific TCR-engineered T cells TCR to target HBsAg-expressing HCC cells;^135^ this platform enables the testing of a wide variety of immunotherapy strategies and allows us to control changes in oxygen level, cytokine administration and/or changes in the TME^92^ cellular composition [Fig. 2(d)]. Koh *et al.* used the same 3D microfluidic model to monitor the targeting of HBV-associated HepG2 cells by engineered TCR-T cells and found that TCR-T cells pre-activated with anti-CD3 beads produced more granzyme and perforin and were better at lysing hepatocytes.^89^ Building up the cellular complexity, Lee *et al.* included the myeloid component of the *in vivo* intrahepatic immunosuppressive TME to test the inhibitory effect of monocytes on engineered TCR-T cells and the combination of PD1/PDL1 blockade with the engineered TCR-T cell.^108^ Remarkably this immunosuppressive effect was specific to the 3D microfluidic coculture and was not observed in the standard 2D cocultures experiments performed *in vitro*, pointing to the capability of this device to mimic the different characteristics of the liver cancer TME. A similar TME model was used to probe the killing of HBV-associated HCC by TCR-T cells after removing endogenous TCR using CRISPR.^136^ Another combination approach was tested by Hafezi *et al.* that demonstrated how the immunosuppressive drugs, Tacrolimus and Mycophenolate Mofetil (MMF), together with TCR-T cells reduced TCR-T cell function. This inhibitory effect was reverted by the transient overexpression of mutated variants of calcineurin B (CnB) and inosine-5′-monophosphate dehydrogenase (IMPDH) in the T cells.^137^ Overall, since their recent adoption, 3D microfluidic devices have shown to be useful *in vitro* models for the rapid testing of cell therapies, as they are easy to use yet able to replicate some of the obstacles of the tumor microenvironment, such as migration through dense ECM and encountering immunosuppressive cytokines and cell types.

Microfluidic tools can also be scaled up to create high-throughput assays to study immunotherapy. This was the motivation behind the development of the CACI-IMPACT platform. The platform consists of multichannel microfluidic devices that are arrayed in a 96 well plate format, and allows cancer cells to be embedded in a 3D extracellular matrix while cytotoxic cell types such as NK cells are introduced in a separate channel. The spatiotemporal dynamics of cytotoxic cell migration and activity can, thus, be monitored with a relatively high-throughput.^138^ This promising technology could potentially be fully automated, and be implemented for preclinical screening of new chemo-, immuno- and cell-based therapies for solid tumors.

Another key development in complex 3D *in vitro* models that is vital for the modeling of HCC is the incorporation of a functional vasculature. *In vitro* vascularization in microfluidic devices can be formed by seeding endothelial cells in dedicated channels, or by taking advantage of the self-organization characteristics of endothelial cells under pro-vascularization conditions, such as with the co-culture of fibroblasts.^139,140^ Fibroblasts can be reprogrammed by cancer cells to remodel the TME, and the coculture of liver cancer spheroids and fibroblast may result in a more immunosuppressive microenvironment.^141^ The vascularization of spheroids/organoids can then be achieved through the co-culture with these with *in vitro* vasculature models.^142^ The *in vitro* vasculature stimulates tumor growth and tumor-associated vascularization and allow for the study of tumor-vasculature interactions and cancer cell intravasation or extravasation. Vascularized organoids/spheroids mimicking *in vivo* flow conditions impact the interactions between cells and the TME, and allow the observation of cancer cell migration, intravasation and proliferation under physiological flow conditions.^143^ Nashimoto *et al.* generated self-organised perfusable vasculature using tumor spheroids co-cultured with fibroblast and endothelial cells in microfluidic devices; in this study, they demonstrated that the vessel-like structures can be used to administer biological substances (i.e., drugs) to the interior of the spheroid and this vascularized tumor model can be used for studying drug efficacy and tumor proliferation.^144^ The development of human tumors-on-chip with integrated perfusable vasculature is useful for studies on drug delivery and how the tumor affects vasculature formation and their impact in the TME^93,143,144^ [Fig. 2(e)]. Although these models have not yet been applied to the study of cell therapies, they would be useful for studying the mechanism by which various factors in the TME impact tumor-vasculature interactions and the targetting of anti-tumor cells. These models would also be useful to study the mechanism and efficacy of anti-angiogenesis drugs currently being administered in clinics and their impact on the TME, and if they could be used as adjuvants in cell therapy regimes.

## V. CONCLUSION AND PERSPECTIVE

In the past decade, with the help of improved microfluidic device design, cell culture techniques, and ECM development, significant advances have been made to create *in vitro* cell culture models and systems that recapitulate important aspects of the complex TME. However, the challenges posed by the TME are intricate to fully replicate *in vitro*, especially considering the intra- and inter-tumor heterogeneity.^35^ Liver cancer heterogeneity depends on the mutational load and underlying risk factors but also on the composition of infiltrated immune cells or tumor infiltrating lymphocytes (TILs). Given the heterogeneity of HCC and complexity of its TME, an *in vitro* model able to simultaneously capture all aspects of it is unlikely. However, complex *in vitro* models that mimic key aspects of the HCC TME will significantly aid the discovery of crucial cell–cell interactions and will serve as important tools for cell therapy validation. These “tumors-on-a-chip” can be recreated in microfluidic devices, which have the potential for scaling-up and high-throughput analysis, leading to potentially novel molecular insights and drug and cell therapy discoveries. In addition, these models could incorporate patient-derived organoids and biopsies, speeding up patient sub-typing and the development of personalized therapies.

The immune cell subpopulation is a critical component of the liver TME, but few published *in vitro* models have incorporated immune cells such as T cell and macrophages thus far, and the usage of a wider population of immune cells remains overlooked. The vasculature around and within the tumor are also extremely important, and models that are able to incorporate it will not only be able to study the interaction between tumor and vasculature but also study the effects of flow, tumor intravasation, and immune cell homing. Recent approaches to create immune-competent tumor spheroids/organoids within an ECM, with or without vasculature, lay important foundations for creating even more physiologically relevant *in vitro* models. Given the diverse mechanisms that liver cancer uses to evade immune cells and the promise of immunotherapy, complex 3D models that recapitulate features of the TME involved in anti-tumor immunity by including immunosuppressive cytokines and cell types, dense ECM and vasculature, will aid in the fine-tuning of existing cancer immunotherapy options and pre-clinical testing of novel cell therapy options.

As complex models develop, it will be important to compare and validate these models against *in vivo* tumors to ensure that they are meaningful preclinical models. The current “gold standard” for preclinical testing remains animal models. However, animal models carry with them ethical concerns as well as technical issues regarding the physiology of animals vs humans, especially when it comes to immuno-oncology. More complex and physiologically relevant *in vitro* models using human or even patient-derived cell lines may aid in therapy validation by providing an additional humanized preclinical model. This, in turn, will improve the likelihood of success in more traditional and costly preclinical animal models.

In conclusion, the complex spatiotemporal relationship between cancer, stroma, and immune cells in liver cancers is increasingly being recapitulated in 3D *in vitro* models. These models will provide important insights into understanding these interactions and serve as useful and more physiologically relevant tools in preclinical functional assays of novel cell therapies. These complex *in vitro* models show great promise to becoming fundamental tools that can be widely-adopted for the discovery of novel targets, therapy development, and validation, especially in the area of personalized medicine.

## Data Availability

Data sharing is not applicable to this article as no new data were created or analyzed in this study.
